# Missed nursing care in surgical care– a hazard to patient safety: a quantitative study within the inCHARGE programme

**DOI:** 10.1186/s12912-024-01877-1

**Published:** 2024-04-07

**Authors:** Katarina Edfeldt, Lena Nyholm, Eva Jangland, Anna-Karin Gunnarsson, Camilla Fröjd, Anna Hauffman

**Affiliations:** Dept of Surgical Sciences, Nursing Research, Uppsala University, Uppsala University Hospital, SE-751 85 Uppsala, Sweden

**Keywords:** Missed nursing care, Reasons for missed nursing care, Surgical care, Patient safety, Fundamentals of Care, Quantitative study

## Abstract

**Background:**

Missed nursing care occurs globally, and the consequences are severe for the patients when fundamental care needs are not fulfilled, nor delivered in a person-centred way. This study aimed to investigate the occurrence and cause of missed nursing care, and the relationship between registered nurses’ and nursing assistants’ perceptions of missed nursing care, in a surgical care context.

**Methods:**

A quantitative study was performed using the MISSCARE survey, measuring missed nursing care and associated reasons, in three surgical wards with registered nurses and nursing assistants as the participants (*n* = 118), during May-November in 2022. The MISSCARE survey also covers background data such as job satisfaction and intention to leave. The survey was distributed paper-based and the response rate was 88%.

**Results:**

Aspects of nursing care rated to be missed the most were ‘attending interdisciplinary care conferences’, ‘turning patient every 2 h’, ‘ambulation 3 times per day or as ordered’, and ‘mouth care’. Differences between registered nurse and nursing assistant ratings were detected for eight out of 24 items, where registered nurses rated more missed nursing care. The uppermost reasons for missed nursing care were ‘inadequate number of staff’ and ‘unexpected rise in patient volume and/or acuity on the unit’. Registered nurses and nursing assistants rated differently regarding six of 17 items. Almost every fourth staff member (24.6%, *n* = 29) had the intention to leave within a year in the present department.

**Conclusions:**

The occurrence of missed nursing care is frequent in the surgical context, and in combination with a high number of staff members intending to leave their employment, poses a hazard to patient safety. Registered nurses, holding higher educational levels, reported more missed care compared with the nursing assistants. The main reason for missed nursing care was an inadequate number of staff. These findings support a warranted investment in nursing within the organisation. The results can be used to form strategies and interventions, to reduce nurse attrition and optimise competence utilisation, and to achieve safe person-centered fundamental care.

**Supplementary Information:**

The online version contains supplementary material available at 10.1186/s12912-024-01877-1.

## Background

Missed nursing care (MNC) occurs globally and is defined as any aspect of required patient care that is omitted or delayed [1]. The consequences of MNC are severe for patients [2]; in addition, MNC has been related to nursing staff levels [3]. Due to an aging population and increased possibilities to treat diseases that previously had a rapidly fatal course, there are more people requiring care, and the health care system is facing major challenges in terms of the availability and quality of care. The World Health Organization estimates that 13 million registered nurses (RNs) need to be recruited within the coming decade [4, 5]. At the same time, the turnover of RNs is high, due to high levels of burnout, work-related stress and dissatisfaction [6]. The shortage of RNs, together with other factors, e.g. high turnover, is forcing RNs to ration their caring responsibilities, leading to task-oriented, fragmented nursing care that results in deficiencies in care [7]. These circumstances are evident in the surgical care context, as the patients are often frail and elderly with multiple illnesses and complex care needs [8]. The European research programme RN4Cast showed a link between shortages of RNs and patients’ risk of injury and postoperative mortality [6]. Several studies confirm this result; where MNC is associated with inpatient mortality following common surgical procedures [2], MNC increases in association with the number of patients an RN is responsible for [9], and the risk of death increases when the nurse staffing level is low [10]. Besides the increased risk of mortality, patients’ well-being and comfort are also negatively affected when fundamental care is not fulfilled; e.g. oral hygiene, skin care, pain management, and comforting [2, 8, 11]. In Sweden, as well as in many other countries, the nursing staff comprises different groups of nurses; RNs and nursing assistants (NAs), with different educational levels and work tasks. RNs are registered health care professionals, most often with a university Bachelor’s degree, while the competence needed for NAs is not regulated and work tasks can differ widely. Commonly, NAs perform patients’ fundamental care needs under the supervision of RNs [12].

To describe the need for a holistic nursing approach and patients’ fundamental care needs, and in response to the increased MNC, the Fundamentals of Care framework was developed [13, 14]. The conceptual framework includes three dimensions: establishing the relationship, the integration and fulfillment of a patient’s physical and psychosocial care needs, and the nurse-patient relationship to recognise and manage these needs. The final dimension refers to the context of care. In the framework, the attention to the context of care (i.e. resources, organisation, leadership, policy) is described as vital to ensure patient safety, and to understand the reasons behind failure in fundamental care delivery. The framework can be used by nursing leaders and staff as a tool in daily practice to identify patients’ individual care needs. Furthermore, the framework has been suggested for use in reinforcing nursing leadership and in discussions of what is needed at an organisational level to achieve fundamental person-centered care [13].

This study is a part of the inCHARGE (Innovations to utilise nurses’ competence and achieve person-centred care– Fundamentals of Care goes into practice) programme, an action research programme with a collaboration between the surgical department in Uppsala university hospital and a research group at Uppsala university. inCHARGE was initiated by the research group at Uppsala University, including registered nurses with a Ph.D. degree, and is led by EJ. The programme has an interdisciplinary focus, integrating the perspectives of nursing science and medical humanities. The overall aim of the inCHARGE programme is to design innovations to retain RNs and to optimise nursing competence utilisation as a means to improve patient care.

This study aimed to investigate the occurrence and cause of MNC, and the relationship between registered nurses’ and nursing assistants’ perceptions of MNC, in a surgical care context.

## Methods

### Recruitment and setting

The study was approved by the Swedish Ethical Review Authority (DNR 2022-01557-01) and registered in a public study register (Public360 DNR 2023-00042). Ethical principles were followed carefully using the provisions of the Declaration of Helsinki. The participants were informed verbally and in writing that participation in the study was voluntary, and that they could withdraw from the study without explanation or consequence. Written consent was collected from all participants.

The data collection was undertaken during May to November in 2022 at the surgical department of a university hospital in Sweden, comprising three care units admitting patients due to vascular, endocrine, colorectal, oesophageal/ventricle, or liver/bile/pancreatic illness, trauma or transplantation. Admission could be both acute and planned. Each care unit had a ward manager and two assistant ward managers responsible for nursing care and staffing. Patients were always cared for by one RN and one NA. The RNs and NAs worked day, evening or night shifts and cared for 5–12 patients, depending on the time of day and the number of patients requiring hospital care. Due to the department being short-staffed by about 20% of the number of RNs needed (in 2022), the patients were also cared for by agency RNs hired for shorter periods. Still, the care units had beds closed for admission due to a persistent shortage of RNs.

There were 134 eligible staff members (RNs *n* = 70, NAs *n* = 64) (agency RNs excluded) who were invited to participate in conjunction with staff meetings and the surgical departments’ routine educational programme for all RNs and NAs. Informed consent was provided by 118 staff members, and the response rate was 88%.

### Data collection

The validated Swedish version of the MISSCARE survey [14, 15] was used to measure how often (MISSCARE part 1), and why (MISSCARE part 2) staff were not able to perform different nursing care measures. In part 1, the respondent replied to 24 different nursing measures (e.g. mouth care, patient education), and answers were given on a five-point Likert scale from ‘always carried out’ to ‘never carried out’. In part 2 the respondent replied to 17 possible reasons why the nursing care was not provided (e.g. inadequate staffing). Answers were given on a four-point Likert scale from ‘significant cause’ to ‘not a cause’. The MISSCARE survey also covered background data such as job satisfaction and intention to leave. The survey was distributed paper-based.

### Analytical strategies

All analysis was performed in SPSS version 28. All staff outcomes were analysed in three populations; one group containing data from all staff (RNs and NAs), one group containing data from RNs, and one group containing data from NAs. When a participant had more than 50% missing values in a questionnaire listwise deletion was performed. All scores were processed according to instructions for the instrument, and the Mann-Whitney U-test was performed to identify differences between groups. A *p*-value of < 0.05 was considered significant.

In addition (and provided in Supplementary Tables 1 & 2) data in the MISSCARE survey was analysed dichotomously and ranked [15, 16] thus, in the first part of the questionnaire (missed nursing care measures) the alternatives ‘occasionally’, ‘frequently’ and ‘always’ were classified as MNC. In the second part (reasons for missed nursing care), the alternatives ‘significant’ and ‘moderate’ were classified as reasons for MNC.

## Results

The vast majority of staff were women with permanent employment, and the mean age was 35 years (min-max: 20–64 years). There was almost an equal distribuation between RNs (53.4%, *n* = 63) and NAs (44.9%, *n* = 53) (missing data; 1.7%, *n* = 2), and most RNs (86.0%, *n* = 55) had a Bachelor’s degree or a one-year Master’s degree in nursing science. See Table 1 for staff characteristics.

**Table 1:** Staff characteristics (RNs and NAs) in the three surgical care units (*n* = 118)

Characteristic	*n* (%)*
Gender	
Female	106 (89.8)
Male	9 (7.6)
Age	
20–30 years	56 (50.5)
31–50	40 (36.0)
51–75	15 (13.5)
Employment	
Permanent employment	117 (99.2)
Hourly employee	1 (0.8)
Professional role	
Registered nurse	63 (53.4)
Nurse assistant	53 (44.9)
Academic degree for RNs	
None	6 (9.4)
Bachelor	51 (79.7)
One-year Master	4 (6.3)
Licenciate	1 (1.6)
Doctor	1 (1.6)

* When numbers in a category do not add up to n or 100% there is missing data. Abbreviations: RNs. Registered Nurses; NAs. Nursing Assistants

RNs’ and NAs’ aspects regarding work experience and satisfaction with the current work situation are presented in Table 2. More NAs (37.5%, *n* = 19) had work experience > 10 years in their role compared with RNs (10.9%, *n* = 7), and they had been employed at the current unit for the same amount of time. RNs (89.1%, *n* = 57) had more hours of overtime than NAs (68.7%, *n* = 35), but more NAs (15.7%, *n* = 8) had > 6 days/shifts absent in the past three months than RNs (1.6%, *n* = 1). More RNs (67.2%, *n* = 43) were satisfied or very satisfied with their current position than NAs (19%, *n* = 25). There was no difference in intention to leave, perceived adequacy of staffing, and satisfaction with profession or satisfaction of the teamwork between RNs and NAs. Almost every fourth staff member (24.6%, *n* = 29) had the intention to leave within a year. The majority of staff (68.7%) perceived that adequate staffing was obtained 50% or less of the time, and 80.1% were satisfied or very satisfied with the team work.

**Table 2:** Aspects of work experience and satisfaction with current work situation (the MISSCARE survey) and differences between registered nurses and nursing assistants

	All	RNs	NAs
	*n* (%)	*n* (%)	*n* (%)
**Experience in the role**			
< 6 months	10 (8.5)	9 (14.1)	1 (2.0)
> 6 months– 2 years	16 (13.6)	9 (14.1)	7 (13.7)
> 2 years– 5 years	40 (33.9)	23 (35.9)	16 (31.4)
> 5 years– 10 years	20 (16.9)	12 (18.8)	8 (15.7)
> 10 years	31 (26.3)	7 (10.9)	19 (37.5)
**Experience at current unit**			
< 6 months	12 (10.2)	9 (14.1)	3 (5.9)
> 6 months– 2 years	23 (19.5)	12 (18.8)	10 (19.6)
> 2 years– 5 years	43 (36.4)	22 (34.4)	18 (35.3)
> 5 years– 10 years	23 (19.5)	11 (17.2)	11 (21.6)
> 10 years	14 (11.9)	6 (9.4)	7 (13.7)
**Hours of overtime in past 3 months**			
None	22 (18.6)	3 (4.7)	16 (31.4)
1–12 h	56 (47.5)	33 (51.6)	21 (41.2)
More than 12 h	40 (33.9)	24 (37.5)	14 (27.5)
**Days or shifts absent in past 3 months**			
None	48 (40.7)	27 (42.2)	16 (31.4)
1 day/shift	13 (11.0)	9 (14.1)	4 (7.8)
2–3 days/shifts	29 (24.6)	14 (21.9)	15 (29.4)
4–6 days/shifts	16 (13.6)	8 (12.5)	8 (15.7)
> 6 days/shifts	11 (9.3)	1 (1.6)	8 (15.7)
**Intention to leave**			
< 6 months	16 (13.6)	11 (17.2)	3 (5.9)
< 1 year	13 (11.0)	4 (6.3)	8 (15.7)
Not < 1 years	88 (74.6)	45 (70.3)	39 (76.5)
**Perceived adequacy of staffing**			
100% of time	5 (4.2)	1 (1.6)	4 (7.8)
75% of time	31 (26.3)	17 (26.6)	13 (25.5)
50% of time	46 (39.0)	23 (35.9)	21 (41.2)
25% of time	27 (22.9)	14 (21.9)	10 (19.6)
0% of time	8 (6.8)	5 (7.8)	2 (3.9)
**Patients in charge of at last shift**			
0 patients	21 (17.8)	6 (9.4)	13 (25.5)
4–6 patients	73 (61.8)	42 (65.6)	28 (54.9)
7 or more patients	24 (20.2)	12 (18.8)	10 (19.6)
**Satisfication with current position**			
Very satisfied	20 (16.9)	13 (20.3)	5 (9.8)
Satisfied	52 (44.1)	30 (46.9)	20 (9.2)
Neutral	29 (24.6)	8 (12.5)	19 (37.3)
Dissatisfied	13 (11.0)	7 (10.9)	5 (9.8)
Very dissatisfied	1 (0.8)	1 (1.6)	0 (0)
**Satisfication with profession**			
Very satisfied	61 (51.7)	40 (63.5)	16 (31.4)
Satisfied/neutral	53 (44.9)	19 (29.7)	34 (66.7)
Dissatisfied	2 (1.7)	0 (0)	0 (0)
Very dissatisfied	0 (0)	0 (0)	0 (0)
**Satisfication with teamwork on current unit**			
Very satisfied	28 (24.1)	15 (23.4)	11 (21.6)
Satisfied	65 (56.0)	36 (56.3)	28 (54.9)
Neutral	18 (15.5)	6 (9.4)	9 (17.6)
Dissatisfied	4 (3.4)	1 (1.6)	3 (5.9)
Very dissatisfied	0 (0)	0 (0)	0 (0)

NOTE: When numbers do not add up there are missing values.

Abbreviations: RNs. Registered Nurses; NAs. Nursing Assistants

### MISSCARE survey

Aspects of nursing care that were rated to be missed the most (ranks 1–3) were ‘attending interdisciplinary care conferences’, ‘turning patient every 2 h’, and ‘ambulation 3 times per day or as ordered’. Differences between RN and NA ratings were detected for eight out of 24 items. The greatest differences were detected for ‘medications administered within 30 min before or after scheduled time’, ‘mouth care’, and ‘emotional support to patient and/or family’. For the first of the previously mentioned items, NAs ranked higher (rank 3) than RNs (rank 11), although many NAs did not answer that question, thus there were a lot of missing data. For all other items that were rated differently between RNs and NAs, RNs rated higher– with therefore more missed nursing care– than NAs, e.g. ‘mouth care’, ‘emotional support to patient and/or family’, and ‘wound care’. MNC is ranked and presented for all staff, RNs and NAs in Table 3. Overall, there was no difference between RNs and NAs in rating MNC (*p* = 0.33). Dichotomised data analysis resulted in the same five items mostly missed and are presented in Supplementary Table 1.

**Table 3:** Missed nursing care measured by the MISSCARE survey part 1

			All				RN				AN			
Nursing care elements		n¹	Mean (SD)	Rank		n¹	Mean (SD)	Rank		n¹	Mean (SD)	Rank		*p*-value *RN vs. AN*
Attend interdisciplinary care conference whenever held		103	3.08 (1.14)	1		60	3.20 (1.15)	1		38	2.97 (1.03)	2		0.27
Turning patient every 2 h		116	3.07 (1.06)	2		60	3.18 (1.0)	2		50	3.02 (1.06)	1		0.50
Ambulation 3 times per day or as ordered		113	2.75 (0.68)	3		60	2.82 (0.68)	3		47	2.70 (0.69)	4		0.33
Assess effectiveness of medication		101	2.50 (0.88)	4/5		60	2.67 (0.77)	5		36	2.28 (1.0)	7		< 0.05*
Mouth care		114	2.50 (0.73)	4/5		60	2.73 (0.69)	4		49	2.18 (0.64)	10		< 0.001*
Medications administered within 30 min before or after scheduled time		92	2.41 (0.94)	6		60	2.20 (0.66)	11		27	2.89 (1.19)	3		< 0.01*
Patient discharge planning and teaching		109	2.35 (0.94)	7		60	2.47 (0.93)	7		44	2.20 (0.95)	9		0.10
IV/central line site care and assessments according to hospital policy		102	2.30 (0.89)	8		60	2.35 (0.80)	8		38	2.24 (1.05)	8		0.27
Emotional support to patient and/or family		116	2.23 (0.86)	9		60	2.48 (0.81)	6		51	1.94 (0.86)	14		< 0.01*
PRN medication requests acted on within 15 min		95	2.20 (0.82)	10		60	2.08 (0.53)	12		31	2.45 (1.18)	5		0.34
Focused reassessments according to patient condition		113	2.13 (0.80)	11/12		60	1.98 (0.80)	14/15		48	1.98 (0.76)	12/13		0.06
Patients teaching about procedures, tests, and other diagnostic studies		114	2.13 (0.72)	11/12		60	2.23 (0.81)	9		49	2.02 (0.72)	11		0.12
Patient bathing/skincare		115	2.12 (0.60)	13		60	2.23 (0.53)	10		50	1.98 (0.65)	12/13		< 0.05*
Feeding patient when the food is still warm		110	2.11 (0.95)	14		59	1.95 (0.73)	16		46	2.37 (1.14)	6		0.07
Assist with toileting needs within 5 min of request		115	1.98 (0.65)	15		60	2.07 (0.58)	13		49	1.88 (0.70)	15		0.09
Patient assessments performed each shift		115	1.86 (0.80)	16		50	1.74 (0.75)	20		50	1.74 (0.75)	18		0.12
Full documentation of necessary data		116	1.83 (0.64)	17		59	1.98 (0.66)	14/15		50	1.64 (0.60)	19		< 0.01*
Response to call light is initiated within 5 min		116	1.79 (0.65)	18		60	1.80 (0.61)	19		50	1.80 (0.73)	16		0.81
Wound care		116	1.72 (0.54)	19		60	1.88 (0.49)	17		50	1.54 (0.54)	21		< 0.001*
Monitoring intake/output		116	1.71 (0.64)	20		60	1.85 (0.66)	18		51	1.55 (0.61)	21		< 0.05*
Setting up meals for patients who feed themselves		111	1.64 (0.96)	21		59	1.54 (0.80)	22		46	1.78 (1.15)	17		0.52
Nursing staffs’ hand washing		116	1.55 (0.66)	22		60	1.63 (0.69)	21		50	1.46 (0.65)	22		0.11
Bedside glucose monitoring as ordered		115	1.47 (0.54)	23		60	1.52 (0.50)	23/24		49	1.43 (0.58)	23/24		0.27
Vital signs assessed as ordered		116	1.41 (0.49)	24		60	1.52 (0.50)	23/24		49	1.43 (0.58)	23/24		0.27

NOTE: The ranking of reported most missed [1] to least missed [21] nursing care elements. When rank is the same for two nursing care elements they received the same mean score. Answers are given on a five-point Likert scale from 1 - “always carried out to 5 - “never carried out”. Abbreviations: RNs.Registred Nurses; NAs. Nursing Assistants. ¹When numbers do not add up there is missing data

The uppermost reasons for MNC were ‘inadequate number of staff’ and ‘unexpected rise in patient volume and/or acuity on the unit’. RNs and NAs rated differently regarding six of 17 items, with the greatest difference regarding ‘heavy admission and discharge activity’, where RNs ranked this as a higher reason for MNC than NAs did. Smaller, but significant, differences were detected for ‘lack of back-up support from team members’, ‘inadequate hand-off from previous shift or sending unit’, ‘supplies/equipment not available when needed’, ‘supplies/equipment not functioning properly’, and ‘other departments did not provide the care needed’. Reasons for MNC are ranked and presented for all staff, RNs and NAs in Table 4. Dichotomised data analysis resulted in thame four items for the strongest reasons for MNC and are presented in Supplementary Table 2.

**Table 4:** Reasons for missed nursing care measured by the MISSCARE survey part 2

			All				RN				AN			
REASONS FOR MNC		n¹	Mean (SD)	Rank		n¹	Mean (SD)	Rank		n¹	Mean (SD)	Rank		*p*-value *RN vs. AN*
Inadequate number of staff		114	1.30 (0.59)	1		60	1.28 (0.59)	1		50	1.30 (0.61)	1		0.92
Unexpected rise in patient volume and/or acuity on the unit		115	1.36 (0.61)	2		60	1,32 (0.57)	2		50	1.38 (0.64)	2		0.65
Urgent patient situations (e.g., patient’s condition worsening)		114	1.86 (0.85)	3		60	1.88 (0.45)	3		49	1.78 (0.87)	3		0.42
Unbalanced patient assignments		114	1.95 (0.81)	4		60	2.03 (0.84)	5		49	1.82 (0.73)	4		0.19
Heavy admission and discharge activity		108	2.19 (0.94)	5		60	2.0 (0.84)	4		43	2.47 (1.03)	11/12		< 0.05*
Tension or communication breakdowns with the medical staff		114	2.22 (0.82)	6		60	2.27 (0.84)	6		49	2.18 (0.78)	5		0.61
NA did not communicate that care was not done		115	2.31 (0.87)	7		60	2.38 (0.87)	7		50	2.24 (0.87)	7		0.34
Lack of back-up support from team members		114	2.40 (0.94)	8		60	2.58 (0.87)	9		49	2.20 (0.96)	6		< 0.05*
Caregivers off unit or unavailable		114	2.48 (0.83)	9		60	2.45 (0.75)	8		49	2.47 (0.94)	11/12		0.85
Tension or communication breakdowns within the nursing team		115	2.53 (0.90)	10		60	2.62 (0.83)	10/11		50	2.40 (0.99)	8		0.17
Medications were not available when needed		101	2.56 (0.84)	11		60	2.62 (0.76)	10/11		37	2.43 (0.96)	9		0.21
Tension or communication breakdowns with other support deparments		114	2.61 (0.83)	12		60	2.70 (0.74)	12		49	2.49 (0.94)	13		0.18
Inadequate hand-off from previous shift or sending unit		115	2.63 (0.80)	13		60	2.80 (0.71)	13		50	2.44 (0.88)	10		< 0.05*
Other departments did not provide the care needed		113	2.79 (0.94)	14		60	2.95 (0.83)	15		48	2.54 (1.03)	15		< 0.05*
Supplies/equipment not available when needed		112	2.80 (0.95)	15		60	2.98 (0.81)	16		47	2.53 (1.06)	14		< 0.05*
Inadequate number of assistive personnel (e.g., NA, techs etc.)		114	2.81 (0.84)	16		60	2.85 (0.78)	14		49	2.71 (0.91)	17		0.33
Supplies/equipment not functioning properly		113	2.91 (0.89)	17		60	3.08 (0.72)	17		48	2.65 (1.02)	16		< 0.05*

NOTE: The ranking of reported strongest reason [1] to least of a reason [17] for missed nursing care elements. Answers are given on a four-point Likert scale from 1 - “significant cause” to 4 - “not a cause”. When rank is the same for two nursing care elements they received the same mean score. Abbreviations: MNC. Missed Nursing Care; RNs. Registred Nurses; NAs. Nursing Assistants. ¹When numbers do not add up there is missing data

## Discussion

Frequently missed nursing care was, according to both RNs and NAs, ‘attending interdisciplinary care conferences’, ‘turning patient every 2 h’, ‘ambulation 3 times per day or as ordered’ and ‘mouth care’. The results indicate that patient safety is lacking, not turning or ambulating patients regularly increases the risk for multiple complications [17], and the lack of oral health care, also stated previously [11], has been associated with hospital-acquired infections and lacking nutritional status, impairing recovery and prolonging hospital stays [18]. These results are similar to previous global research [14, 19–21]. In Iceland [22], the United States and South Korea [19], all of the above-mentioned items were mostly missed, although slightly less missed than our results, except for ‘mouth care’ in the United States, which was missed on the same level as in this study.

Reasons for MNC, according to both RNs and NAs, were ‘inadequate number of staff’, ‘unexpected rise in patient volume and/or acuity on the unit’ and ‘urgent patient situations’. These results are similar to research in another Swedish context [20, 21]. However, internationally, there are studies that report substantially fewer problems with a lack of resources [19, 22]. Interestingly, this problem differs between countries, and further analysis is acquired to understand the cause. However, the work environment needs to improve through authentic leadership to increase job satisfaction, decrease intention to leave, and thereby reduce reasons for MNC [23]. The greatest difference between RN and NA ratings was regarding ‘heavy admission and discharge activity’, where the workload most likely increases more for RNs than NAs due to the increased responsibilities of RNs in these situations.

MNC can be an indicator of a too high patient-nurse ratio, thus a too high workload and inadequate number of RNs. In this study, the majority of staff estimated that staffing was only adequate 50% or less of the time, and the nurse managers stated that the departments were short-staffed by 20% of the number of RNs needed. RNs’ perception of staffing adequacy greatly influenced their intention to leave [24]. A high patient-nurse ratio increases burnout, ethical stress and job dissatisfaction, which in turn leads to an increase in the intention to leave the workplace [25, 26]. In our cohort, as many as 24.6% had the intention to leave within a year, which is in concordance with Rudman et al. (2014) [27] and Nymark et al. (2023) [20], indicating that today and for almost 10 years the problem has remained unsolved. This is an alarmingly high number, and actions must be taken to retain nurses. The World Health Organization states that work satisfaction is one of the most important factors (together with work motivation) for resolving problems with recruiting and retaining staff in the healthcare sector (WHO, 2016). Sufficient numbers of RNs decreases readmission rates and mortality [6, 10]. An acceptable patient-nurse ratio may increase job satisfaction, and the intention to stay, but most importantly it will decrease MNC and increase patient safety.

When studying nursing care, there is a need to separate RNs and NAs based on their different knowledge/educational level and responsibilities, because they perceive and report MNC differently in one-third of the 24 items in the survey. The same pattern has been seen in previous studies [20, 22]. It is the RNs’ responsibility that fundamental care is performed, but some tasks are carried out by NAs. Interestingly, our results show, similar to Nymark et al. (2023) and Bragadottir et al. (2018), that RNs rate the performance of preferably NA tasks (such as ‘patient bathing/skincare’, ‘wound care’, ‘monitoring intake/output’ and ‘mouth care’) higher than NAs rate them, thus more missed. A possible explanation for differences in ratings of MNC can be a lack of communication within the nursing team. Communication within the care team is important for quality of care and patient safety, and for RNs to be able to lead the nursing care [28], and also affects RNs’ intention to leave their position [24]. Our results show that 18.9% of staff were not satisfied (or neutral) with teamwork, thus improvements are warranted. Another reason may be that RNs have a solid education and may therefore have a more comprehensive perspective than NAs, and are able to thoroughly evaluate performed nursing care, and respond to abnormal signs [29]. These results, together with the results from a study by Griffiths et al. (2019), who states that the hazard of death decreased linearly to the amount of RN staffing but increased with more NA staffing, indicate that RN shortages cannot be solved with an increased number of less-trained nursing staff. The context of care has to be considered when analysing MNC. In the organisation, nurse managers are responsible for routines and culture, which are important to achieve good communication within the care team, and also to enable RNs to lead nursing care. Nurse managers also have to articulate the importance of RNs’ competence together with patient safety in policy discussions and decisions.

The reports of MNC within an organisation are a serious issue, and nursing leaders need to respond to and resolve the reasons behind the failure [30]. To decrease MNC and achieve an evidence-based nursing practice, theory-guided interventions are useful [31]. One innovative ongoing effort to improve nursing care in the present department is the InCHARGE programme, which aims at finding solutions to MNC and improving person-centred fundamental care in the surgical setting. The programme is a joint university– university hospital collaboration, where nursing researchers, clinical nurses, nurse managers and leaders collaborate to implement person-centered fundamental care guided by the Fundamentals of Care framework. Kitson et al. (2019) [30] state that fundamental care must hold value and be foundational to all caring activities within an organisation, and the delivery of such care needs to be seen as the minimum standard of care. In an implementation process, the Fundamentals of Care framework can be used in reinforcing nursing leadership, and also in discussions about changes at the organisational level to achieve person-centred fundamental care [13].

A strength of this study is the high response rate of 88%. As the study requested anonymous returns and implied consent, it was not possible to identify staff who did not participate to explore reasons for not participating, nor to analyse the characteristics of non-respondents. A limitation was that the study is performed in only one hospital. Another strength is that the MISSCARE survey is a well-tested tool. However, the items regarding medication, which are specifically an RN’s responsibility, had in our study a lot of missing values for NAs, who might not know if those actions were performed or not, hence not answering the question.

## Conclusions

The occurrence of missed nursing care is frequent in the surgical context, and in combination with a high number of staff members intending to leave their employment, poses a hazard to patient safety. Registered nurses, holding higher educational levels, reported more missed care compared with the nursing assistants. The main reason for MNC was an inadequate number of staff. These findings support a warranted investment in nursing within the organisation. The results can be used to form strategies and interventions, to reduce nurse attrition and optimize competence utilisation, to achieve safe person-centred fundamental care.

### Electronic supplementary material

Below is the link to the electronic supplementary material.

Supplementary Material 1

## Data Availability

The dataset analysed is available on request from the first author.

## Abbreviations

inCHARGE: Innovations to utilise nurses’ competence and achieve person–centred care Fundamentals of Care goes into practice
MNC: missed nursing care
NA: nursing assistant
RN: registered nurse
